# Optimised Convolution Layers of DnCNN using Vedic Multiplier and Hyperparameter Tuning in Cancer Detection on Field Programmable Gate Array

**DOI:** 10.2174/0115734056400656250616073019

**Published:** 2025-06-20

**Authors:** S. Roobini Priya, Prema Vanaja Ranjan, Shanker Nagalingam Rajediran

**Affiliations:** 1 Department of EEE, CEG Campus Anna University, Chennai, Tamil Nadu, India; 3 Department of Computer Science, Aalim Muhammad Salegh College of Engineering, Chennai, Tamil Nadu, India

**Keywords:** Vedic multiplication, Urdhva Tryambakam Sutra, Nikhilam with upasutra, CNN, FPGA, Breast cancer segmentation

## Abstract

**Introduction:**

Recently, deep learning (DL) algorithms use Arithmetic Units (AU) in CPU/GPU hardware for processing images/data. AU operates in fixed precision and limits the representation of weights and activations in DL. The problem leads to quantization errors, which reduce accuracy during cancer cell segmentation.

**Methods:**

In this study, arithmetic multiplication in convolution layers is replaced with Vedic multiplication in the proposed DnCNN algorithm. Next, Vedic multiplication-based convolution layers in the DnCNN architecture are optimized using POA (Pelican Optimization Algorithm), and the resulting POA-DnCNN is implemented on an FPGA device for breast cancer detection, segmentation, and classification of benign and malignant breast lesions.

**Discussion:**

In the convolution layer of DnCNN, floating-point operations are performed through the Hybrid-Vedic (HV) multiplier called ‘CUTIN,’ which is the combination of *Urdhva Tryambakam* and *Nikhilam Sutra* with the upasutra ‘*Anurupyena*.’ Larger image sizes increase processor size and gate count.

**Results:**

The proposed HV-FPGA-based breast cancer detection system, employing Vedic multiplication in the convolution layers of DnCNN and hyperparameters optimized by POA, detects stages of breast cancer with an accuracy of 96.3%, precision of 94.54%, specificity of 92.37%, F-score of 93.56%, IoU of 94.78%, and DSC of 95.45%, outperforming existing methods.

**Conclusion:**

The proposed CUTIN multiplier uses a CSA (carry save adder) with simplified sum-carry generation logic (CSCGL), achieving lower area-delay, high speed, and improved precision.

## 1 INTRODUCTION

Early detection of breast cancer increases survival rates in women. Breast cancer [1] is detected based on symptoms; however, many women do not exhibit any symptoms. Breast cancer detection is performed using mammography, ultrasound, magnetic resonance imaging (MRI) images, and biopsy. However, these methods may not have the necessary spatial resolution to clearly identify small tumors, particularly in the early stages of breast cancer. Mammogram images are used for breast cancer detection based on masses and micro-calcifications in image classifications using deep learning algorithms, such as RSNA [2]. RSNA datasets lack enough examples of early-stage cases, which restricts the model's ability to generalize to and accurately identify types of tumors. Machine learning (ML) techniques are used to enhance breast cancer detection and prognosis using Support Vector Machines (SVM) [3]. In breast cancer segmentation, the relationship between image features and tumor boundaries is often complex and may require careful tuning of the SVM, which can be computationally expensive and difficult to optimize. AI and computer vision-based state-of-the-art technologies and algorithms have been proposed for the identification of breast cancer using multimodal imaging. Mammogram images [4] are used for the initial screening of breast cancer. ConvNet-based deep learning algorithms [5], CNN algorithms [6], and You Only Look Once (YOLO) v3, CNN-SVM [7], and transfer learning [8] predict breast cancer [9] and correlate the findings with histopathology images. The computational architectures of current deep learning algorithms [10] use AU in the convolution layer. Appropriate computing techniques are employed, such as weights, activation functions, and biases in deep learning methods, which need to be selected for accurate segmentation. LOCO, a fast convolutional computing AI chip, is used [11] for the acceleration of convolutional computation with minimized area and power. Deep learning (DL) [12] based AI algorithms are used for the detection of breast cancer in digital mammography images using ChOA optimization, which has premature convergence to local optima problems, especially in complex or high-dimensional search spaces. DL model [13] shows promising results in breast cancer detection techniques that are useful in error-resilient image processing. In this article [14], different imaging techniques are reviewed, concluding that the hybrid approach is an acceptable method, although it is limited in many healthcare units due to the expense of operation and maintenance. Table **1** exhibits a comparison of existing multiplier techniques [15-18].

## 2 PROBLEM STATEMENT

Fixed-point arithmetic [19] computes quickly and consumes fewer hardware resources during the implementation of DL algorithms in hardware. Floating-point numbers require rounding methods to reduce quantization errors in each layer of the DL architecture. However, high-precision computations remain a major problem for DL engineers and researchers. This problem can be addressed through proper hardware selection, such as CPU, CPO, TPU, and number systems, such as posit fixed point, floating point, and rounding methods like stochastic rounding and probabilistic rounding. However, multiply-accumulate (MACC) operations are performed using arithmetic multiplication. The accuracy of deep learning is based on the optimization of hardware and software levels. The deep learning weights are computed using low-precision arithmetic multiplication, specifically low-precision [20] fixed-point number systems. Traditional methods apply DL algorithms with the optimization of hyperparameters. Few methods apply hardware-level architectures in FPGA optimization using number systems, such as fixed point and floating-point numbers [21, 22] using 16-bit/32-bit systems. In this study, the DL algorithm for segmentation of breast cancer is employed, followed by hardware-level optimization, to detect breast cancer at an early stage.

### 2.1 Motivation

Deep learning techniques, such as CNNs, have gained significant attention in segmentation and classification. However, these models still need refinement, as the deep features extracted from each patch often fail to capture spatial details for accurate segmentation due to subsampling layers. While numerous methods for hyperparameter tuning of deep learning models are available, achieving high accuracy in breast cancer detection remains challenging. These challenges drive the need to enhance CNN hardware design by incorporating low-bit-width integer representations, using floating-point operations designed with more efficient adders and Vedic multiplier units, which replace traditional AUs in deep learning models.

### 2.2 Contributions

The primary objective of the work is to optimize the design of DnCNN on an FPGA device for mammography segmentation. To develop the DnCNN architecture on an FPGA device by adopting the ‘Vedic Hybrid Multiplier,’ *i.e*., the proposed Vedic multiplier CUTIN based on Carry Save Adder with a ‘Clarified Sum-Carry Generation Logic (CSCGL)’ featuring simplified sum-carry logic, to replace traditional computational elements, such as multipliers.

To determine the optimal compromise between accuracy and complexity, the implementation of DnCNN is based on Vedic floating-point operations using the proposed CUTIN-Multiplier. Moreover, the HV-FPGA devices are analyzed for power, area, time, and hardware complexity with existing algorithms, such as (i) *Urdhva Tryambakam* and (ii) *Nikhilam* with upasutra *Anurupyena* and software-based DnCNN, *i.e*., Python-based simulation.

The main contributions of the proposed method are as follows:

- To apply hardware optimization in FPGA devices for the detection of breast cancer at the earliest stage using the DnCNN algorithm on FPGA devices, optimizing multiplication in convolution layers using Vedic multiplication and referring to the system as the HV-FPGA device.
- To optimize the multiplication in the DnCNN algorithm convolution layer on FPGA devices, Vedic multipliers, such as (i) *Urdhva Tryambakam* (UT) and (ii) *Nikhilam* sutra with upasutra (sub-sutra) “*Anurupyena*” (NAS) are used in MACC by utilizing 16-bit operations of the floating-point number system, thus reducing error and hardware complexity through POA optimization.
- To optimize the DnCNN programs using hyperparameters, the POA-tuned hyperparameter of the DnCNN (‘POA-DnCNN’) algorithm segments the breast cancer image for the earlier detection of breast cancer.

The upcoming sections are structured as follows: Section 1 introduces the existing model, detailing the research gap. Section 2 discusses the problem formulation along with motivation and contribution. Section 3 describes the proposed method in detail, and Section 4 presents the result analysis with discussion. Section 5 focuses on the hardware implementation, and Section 6 summarizes the conclusions of the proposed method, along with future directions.

## 3 PROPOSED SYSTEM FLOW

In the Computer Aided Diagnosis (CAD) system, image segmentation is a crucial task. This study proposes DnCNN for the segmentation of tumor cells in MRI breast images with hardware and software-level optimized CNN. However, due to adders supporting multiple operands and floating-point representations required for operations and storage procedures, the DnCNN structure takes up more space. To address this problem, this study presents a streamlined hardware design for CNN that uses an optimized adder and multiplication unit. Additionally, incorrect architecture configuration parameter selections (weight parameters) impact the performance of any deep learning network. Fig. (**1**) illustrates the workflow of the proposed DnCNN model.

### 3.1 DnCNN Hyperparameter Tuning

DL techniques are used for the automatic identification of breast cancer lesions. Hence, it is difficult to differentiate normal cells from cancer lesions since the morphological and textural characteristics of lesions are identical to those of healthy cells. The lack of pathological information makes it difficult to differentiate cancer cells. As a result, several optimization techniques are used for distinction. In this study, DnCNN is optimized with PSO (Particle Swarm Optimization), Bayesian Optimization Algorithm, and Pelican Optimization Algorithm (POA). The need for systematic hyperparameter tuning is a key research gap in the field of DL-driven breast MR image segmentation. Hyper-parameters, such as threshold values, kernel size, and other parameters, create a significant effect on DL models. Hence, effective parameter tuning of DL algorithms that are specifically designed for the task of breast cancer segmentation analysis is proposed. Eliminating this gap is important to achieve top-tier precision and reliability in breast cancer detection. Hence, this study proposes a Pelican Optimization Algorithm (POA) for DnCNN, which is referred to as ‘POA-DnCNN’.

### 3.2 Proposed ‘POA-DnCNN’

The POA is a population-based method inspired by the behavior of pelicans, with each individual in the population representing a pelican. Each member of a population in a population-based algorithm is a possible solution. Based on their position in the search space, each individual of the population proposes a value for the variables in the optimization problem. The lower and upper limits of the problem are used to initially determine random population members.

**Table:** 

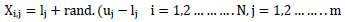	(1)

Where the value of the parameter is denoted by the solution of the candidate. N denotes the size of the population, m is the number of factors in the problem, and rand is a random number within the interval [0,^1^], while l_j_ and u_j_ denote the lower bound and upper bound of the problem variables, respectively. The proposed POA-DnCNN replicates pelican behavior and strategy during prey hunting to update candidate solutions. This hunting method is carried out in two stages: the “Exploration Phase” (‘moving towards prey’) and the “Exploitation Phase” (‘winging on the water surface’). The proposed POA-DnCNN begins with pre-processing (de-noising) and then uses POA to find the most optimal segmentation parameters for DnCNN, followed by post-processing and evaluation. A flowchart of it is presented in Fig. (**2**). The steps involved are as follows:

Steps involved in POA-DnCNN

1. Input is the MRI/ultrasound/mammogram, which contains noise that affects the accuracy of segmentation.

2. CNN layers will be trained to distinguish noise from real image features, making tumor regions apparent for segmentation.

3. POA is initialized for hyperparameter tuning. During this phase, the pelicans explore the space to identify the optimal configuration for DnCNN.

4. *POA optimization:* Pelican (solution) is evaluated by running DnCNN on the denoised image and analyzing segmentation performance. Segmentation quality is measured using a fitness function, such as the Dice Similarity Coefficient (DSC) and Intersection over Union (IoU). Fitness relies on segmentation quality, lesion distinction accuracy (malignant *vs*. benign), and tumor boundary clarity after denoising.

Exploration Phase: *Pelicans explore the solution space for better segmentation and lesion differentiation.* Exploitation Phase: After finding an ideal collection of hyperparameters, pelicans move toward it and improve segmentation accuracy. Cooperation: Pelicans share the best solutions, allowing the population to find the optimal hyperparameter configuration faster. Segmentation: After POA converges to the optimum hyperparameters, DnCNN segments. Based on optimum settings, the network will segment the tumor from adjacent tissue. A segmentation mask from the DnCNN will indicate the lesion's ROI (Region of Interest). Lesion Differentiation (Malignant *vs*. Benign): After segmenting the lesion, malignant is distinguished from benign lesions that include shape, texture, and intensity. Classification: Based on retrieved features, a classifier classifies the lesion as malignant or benign, and this paper proposed a classifier, ‘P-SVM’. The classifier helps radiologists diagnose malignant and benign lesions using the above-mentioned features. Post-processing: Morphological post-processing methods, such as dilation and erosion, can improve segmentation, eliminate noise, and smooth lesion borders. The final segmented image will feature a visible tumor boundary. The outcome is a segmented breast image with clearly marked tumors and a malignant or benign diagnosis. This enables the calculation of metrics, such as the Dice Similarity Coefficient (DSC), Intersection over Union (IoU), sensitivity, and specificity that are used to assess segmentation performance.

Table **2** demonstrates the architecture of DnCNN, and the convolution kernels of DnCNN are used for multiplication and addition of three-dimensional input feature maps. Input features are convoluted with the kernel weights to obtain the output feature map. The outputs are transmitted to the next level of the adder tree after multiplication, where addition is done on the intermediate results, as illustrated in Fig. (**3**). The convolution operation needs frequent switching among input characteristics and convolution kernels, with significant data access.

### 3.3 Methodology

Computation in the DnCNN accelerator design is carried out by the processing element (PE) unit. Convolution of the input feature map with kernel weights in the PE generates one pixel in the output feature map. Various ways of designing the DnCNN accelerator are explored, which reduces hardware implementation costs. The optimization process involves replacing conventional arithmetic units with the proposed *‘HV multiplier and adders’* realization. The proposed DnCNN quantizes its feature map into a lower bit-depth representation and performs floating-point calculations, achieving the best results through quantized convolution. Symmetric quantization is used for floating-point representation, while DnCNN employs batch normalization for faster training, with ReLU activation to mitigate vanishing gradients. The convolutional layers leverage inner vector products and adders to achieve efficient computation. Input features and filter patches are used to derive vectors initially. The output pixels are produced by computing inner vector products between the inputs and filter vectors. The next layer’s output feature maps are generated using a floating-point module. The hardware complexity arises due to floating-point adder and multiplier components, which are replaced with the proposed CSCGL and CUTIN, respectively, in the hardware architecture.

#### 3.3.1 Proposed CUTIN Multiplication

Multiplying ***‘N x N’*** bit numbers requires ***‘N/2 x*** N/2’ bit multipliers. This method of multiplication is performed in parallel with drastically high speed when compared with sequential multiplication. This makes the microprocessor multiplier independent of the clock frequency; as a result, the multiplier needs the same time to compute products and additions simultaneously. The preceding criteria show that Vedic Sutras are built on parallelism and pipelining, and VM design differs from typical multipliers due to its novel approach. Verilog HDL is used to construct the proposed multiplier, utilizing structural modeling effectively and verified for functionality in FPGA. The novel ‘Vedic Hybrid Multiplier’ solves complex computing through two sutras termed CUTIN (combination of Urdhva Tryambakam ‘UT’ and Nikhilam sutra with upasutra Anurupyena ‘NAS’). Combining sutras allows for breaking problems into simpler components for computation, and larger multiplications can be computed simultaneously in parallel blocks. CUTIN implements UT for PP and NAS for complements to accelerate the concurrent operations in FPGA. For better optimization of FPGA resources like LUTs, registers, and arithmetic units, hybrid sutras avoid unnecessary duplication of logic and reduce overall footprint designs. Thus, by using multiple Vedic sutras, it is possible to design hierarchical units that handle different mathematical sub-tasks. *‘CUTIN’* computes the mantissa using a 48-bit multiplication product, given that both operands are 24 bits in length. Since the resulting bit length is twice that of the operands, the normalization process eliminates the extra bit in the mantissa region and adjusts the exponent accordingly. If the leading bit of the mantissa is high, and the next 23 bits are set, the exponent is incremented by one. The mantissa bit from the (n-2) position is considered if the leading bit is zero and the exponent remains unchanged. When the exponent ranges from 1 to 254, the most significant bit (MSB) of the result determines whether the integer is positive or negative.

#### 3.3.2 Urdhva Tryambakam (UT) Sutra

UT, meaning ‘vertical and crosswise,’ is used for multiplication that enables simultaneous synthesis and addition of all partial products. Once the partial products (‘PP’) are developed, they are combined in parallel, handling the carries efficiently using CSA or CLA (Carry Look Ahead adders), speeding up the summation of PP. This type of architecture does not require stages to accumulate PP; instead, it performs a pipelined architecture executing concurrently. Since FPGA hardware can be tailored to exploit parallelism in both the multiplication and addition steps, UT for FPGA multiplication leverages the inherent parallelism of FPGAs and the efficient handling of carries, leading to high-speed multiplication. It reduces numerical strength, and the results indicate a reduction in hardware, leading to a reduction in latency, as shown in Fig. (**4a**) with a line diagram. A 3x3 bit multiplier, with multiplicands ‘A’ and ‘B,’ has three bits for each multiplicand and six bits for the result.

#### 3.3.3 Nikhilam with Upasutra Anurupyena Sutra (NAS)

**Fig. (4):**
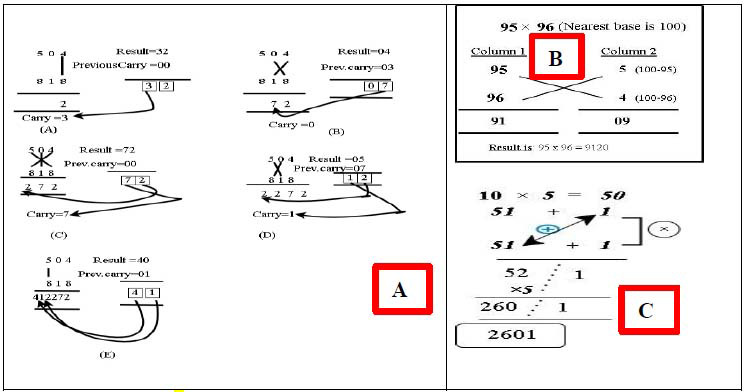
(**a**) Urdhva Tryambakam Sutra, (**b**) Nikhilam Sutra, (**c**) anurupyena sutra manipulation.

In the Nikhilam Sutra, the principle *“All from 9 and the last from 10”* is applied. This method is particularly effective for numbers that are close to common powers of 10, like 10, 100, 1000, etc., or higher powers of 10. It involves using both a theoretical base (powers of 10) and an operational base, which is a multiple of 10, 100, 1000, and so on, for multiplication, as shown in Fig. (**4b**). Numbers can be either greater or smaller than the base value, and their complements are calculated. These complements are then multiplied from the base value to compute the product. Essentially, multiplying two large numbers is the same as multiplying their complements, provided that the complements are not as large as the original numbers. The Nikhilam Sutra is useful when either the multiplicand or the multiplier (or both) is close to a base, making multiplication simpler. However, when the two numbers being multiplied are not near a convenient base, the upasutra “Anurupyena” offers a solution (Fig. **4c**). This upasutra, meaning “proportionately,” suggests that when there is a rational relationship, the ratio should be considered, leading to proportional multiplication based on the situation. To carry out the multiplication with a suitable base, an appropriate ‘working base’ ‘N’ is selected for the operation.

The proposed approach substitutes the adder units in conventional Vedic multipliers with CSCGL, enhancing performance in area, latency, and power. For sum and carry generation, CSCGL features simpler combinational logic. The proposed VM reduces power usage, hardware utilization, latency, and area consumption compared to conventional multipliers. This study presents an 8x8 VM architecture and accelerates the multiplication. If we consider the two 8-bit numbers ‘a’ and ‘b’ as a = and b =, by multiplying the least significant bits (LSBs) a0 and b0, the resultant is LSB s0. The procedure for the 8x8 Vedic multiplier is identical to the 4x4 multiplier, commencing with a zero pre-carry. Each phase involves obtaining carry and adding it to the following stage until the desired output is achieved. The resultant is illustrated in Fig. (**5**). Quantizing a CNN has enormous benefits, as it can reduce the precision of weights, activations, or both. It is a model of optimization to improve computational efficiency since FPGAs are highly customizable for implementing efficient machine learning tasks, including de-noising CNNs. Hence, floating-point arithmetic provides high precision for handling complex computations, as small variations in pixels can significantly impact image quality after de-noising. Fixed-point arithmetic leads to the accumulation of errors over various layers of CNN, resulting in a loss of accuracy, whereas floating-point arithmetic with larger precision helps minimize errors. In a de-noising CNN, input range and activation from different layers vary widely, and floating-point representation varies from very small to large numbers. This is crucial for CNNs, as it involves transformation (such as convolution, activation, and normalization), helping the network process images effectively. Since a de-noised CNN needs to process images with different noise intensities, floating-point representation ensures network adaptability to maintain performance as it covers a wide range. As FPGA allows for parallel execution of operations, FP arithmetic enables highly efficient execution of CNN layers. The quantization architecture of the proposed DnCNN, as shown in Fig. (**6**), comprises a data buffer, processing elements, normalization, activation layers with proposed CUTIN, and CSCGL adder.

#### 3.3.4 CSCGL

The conventional Carry-Save Adder (CSA) is made up of Full Adders (FAs), which serve as its fundamental components. Each FA produces a sum and a carry, propagating the carry to the next stage and saving the sum and carry in the intermediate stages. In a conventional CSA, for *k* N-bit numbers, the result will be *N+1* bits for both sum and carry. Hence, conventional CSAs require additional steps to propagate carries, leading to complexity in multistage operations. The use of multiple full adders increases the hardware area and requires a final carry propagation, potentially limiting the speed of the final result generation. In contrast, the proposed CSCGL (Clarified Sum and Carry Generation Logic) utilizes Carry Select Adders (CSAs) combined with Carry Save Adders (CSA) to accelerate addition speed. The CSCGL differs by quickly selecting the correct carry before the final addition instead of propagating the carry.

**Fig. (7):**
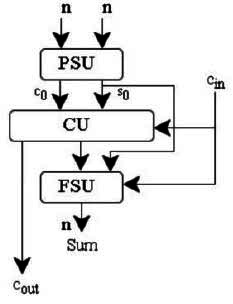
Clarified sum and carry generation.

The enhanced CSA (‘CSCGL’) results in a sum of all operands with minimum carry propagation delay. (Fig. **7**) demonstrates the architecture design of CSCGL, which includes a Partial Sum Unit (PSU), a Final Sum Unit (FSU), and a Carry Generating Unit (CU). In the PSU, the two n-bit entities ‘*A*’ and ‘*B*’ generate the partial sum *s0* and carry as *c0*. The obtained sum *s0* and carry *c0* are provided as input to the FSU. Based on the input carry *cin*, the CU unit chooses the carry, which is combined with the partial sum *s0* to compute the overall sum. Without using a multiplexer, the CU unit is leveraged to strengthen the performance of the system.

## 4 RESULTS AND DISCUSSION

MATLAB and Xilinx tools are used to analyze the performance of the proposed technique as software optimization and hardware optimization. A Numato Lab MIMASV2 Spartan-6 FPGA development kit is used for FPGA implementation. The proposed DnCNN-based segmentation framework makes use of Verilog hardware description language, which includes IC-linked functions and libraries. The discussion is sectioned as “Software Optimization,” “Hardware Optimization,” and “Performance Evaluation” of the proposed DnCNN against various state-of-the-art methods from the literature.

### 4.1 Software Optimization

#### 4.1.1 Breast Cancer Data Description

The significance of the BUSI dataset is to provide support for the classification and detection of breast cancer in ultrasound images. It serves as a basis for the algorithms to be developed, tested, and compared in tumor classification to distinguish between malignant and benign tumors, feature extraction wherein features are extracted from the ultrasound images for accurate diagnosis, and the segmentation of images or isolating the tumor region within the ultrasound image. To train the proposed breast lesion recognition and categorization system, the BUSI mammography dataset is utilized. The original image size is 1024 x 1024, with 133 aberrant and 189 healthy images. Unhealthy images have higher asymmetry mass density, while abnormal instances consist of 21 images. Additionally, structural distortion, a significant anomaly caused by poor venous arrangement, is depicted in 22 images. Calcification, a severe condition, involves decreased calcium storage in the breast, as seen in 24 images. Images of 24 breasts with suspected tissue lumps, deformed borders, and margins are also presented. There are no signs of malignancy present in 20 images. The study employed 312 breast MRI scans using ground truth masks. The dataset is divided into training (252) and testing (60) sets in an 80:20 ratio.

#### 4.1.2 Performance Metrics

Segmentation includes assessment indicators, such as accuracy, precision, F-score, specificity, sensitivity, IoU, and DSC, which are compared with more recent strategies, as presented in Table **2**. TP, TN, FP, and FN correspond to the number of correctly identified positives, correctly identified negatives, misclassified positives, and misclassified negatives pixels, respectively. The performance of the segmentation model improves with larger values of the five metrics. *F-Score* is the combined measure of precision and sensitivity (recall) based on the harmonic mean.

Table **3** summarizes the overall system impact of the proposed DnCNN alongside various models from the literature. The proposed DnCNN performs consistently with the BUSI datasets, proving its breast lesion segmentation durability and adaptability. Among the models evaluated, the DnCNN yields the highest dice coefficient with values of *95.45%.* These superior DSC scores indicate that DnCNN is very accurate in segmenting regions of interest. It proves that it is effective in classifying and contouring breast cancer lesions. The optimal DSC attributes are important because they indicate that the proposed model can improve segmentation accuracy by aligning regions with actual areas of interest and minimizing classification errors. Fig. (**8**) shows that the proposed DnCNN outperforms other DL designs in segmentation metrics. Medical imaging relies on accurate classification to reduce misdiagnosis and ensure proper treatment. However, the accuracy of the proposed DnCNN can be further enhanced through optimization using POA, termed ‘POA-DnCNN,’ and is discussed in the following section.

#### 4.1.3 Analysis and Optimization of the Proposed Tumor Segmentation Using ‘POA-DnCNN’

This proposed DnCNN is optimized using POA-DnCNN with convolution layer breast tumor segmentation strategies using the BUSI dataset. A comparison is shown in Fig. (**9**) for the accuracy test curves of LASSO-BO [23], FO-DPSO [24], RPAOSM [25], BEOSA [26], DnCNN (without POA), and PSO-DnCNN. For the comparison, 45 and 24 training epochs are required to attain the highest accuracy of segmentation. The proposed DnCNN model achieves convergent segmentation in 24 epochs with improved accuracy due to POA parameter optimization and appropriate learning rate. The segmentation outcomes are shown in Figs. (**10**) and (**11a-c**) as image with noise, MATLAB segmented image [27], and de-noised image, respectively, for benign and malignant cancer lesions. The result demonstrated that image segmentation to the original mammography dataset [28] accurately distinguishes ‘benign and malignant’ lesions. A comparison of software, hardware, and performance evaluation reveals that the proposed DnCNN is enhanced due to “Vedic Hybrid Multiplier-” based floating-point operations.

### 4.2 Hardware Optimization

The hardware optimization of CNN can be achieved with reduced speed by using low-bit width integers for activation functions. Hence, the narrow data types are used in the proposed method for the computation of the convolutional layer. Furthermore, floating-point representation is utilized for normalization and activation layers using MATLAB.

#### 4.2.1 FPGA Performance Analysis

The proposed DnCNN is built with a hybrid Vedic multiplier and CSCGL adder that replaces a conventional multiplier and adder design. Table **4** presents the device utilization summary for the proposed multiplier realization and shows that the design occupies and utilizes fewer cells than available. The utilization summary of bits illustrates that the proposed model can be expanded to lower and higher bits.

As mentioned in Table **5**, the proposed HV-multiplier occupies 73.3% less area and has 93.7% less delay in comparison to the Wallace Tree FPM [18], 77.90% less area and 80.1% less delay in comparison to the Booth-based FPM design [16], 92.45% less area and 86.76% less delay when compared to the Dadda FPM design [15], and 84.53% less area and 77.90% less delay as compared to the Array-based FPM [17]. Comparisons reveal that the proposed *HV-FPM (Hybrid Vedic-Floating Point Multiplier)* is more efficient than alternative designs. Using the values of ADPs (Area Delay Product) and PDPs (Power Delay Product), a graph shown in Fig. (**12a**) is drawn with an X-axis representing FPM designs and a y-axis representing ADPs and another graph shown in Fig. (**12b**) is drawn with an X-axis representing FPM designs and a y-axis representing PDPs, showing that the proposed HV-FPM utilizes less area and power compared to alternative designs.

Table **6** provides a summary of the device utilization of the proposed HV multiplier against existing multiplier designs. It demonstrates that the proposed HV-multiplier makes use of fewer occupied cells and IOBs with less propagation delay and time. The implementation results show that the proposed HV multiplier performs with better scalability against other multiplier designs, making it ideal for designing an optimized CNN model.

Table **7** summarizes the hybrid Vedic multiplier combination with different adder topologies and shows that the proposed CSCGL combined with the HV multiplier achieves better specifications, demanding fewer LUTs, power, IOBs, and delay. The adder topology forms the necessary building blocks within the multiplier and propagates the carry in a critical path to compute the sum. Hence, selecting an optimized adder or its combination is a significant step in multiplier design, and the CSCGL achieves it.

### 4.3 Performance Evaluation

This section explains the essential metrics that evaluate the performance in predicting breast cancer lesions and identifying the lesion classification with more accuracy. While evaluating the performance of an ML model, two essential measures, the accuracy of training and validation, play a vital role. Table **8** summarizes the training accuracy and validation accuracy of the proposed FPGA-based CNN (‘DnCNN’) and the non-FPGA-based CNN from the literature. These architectures are named as follows: GUI [1], RSNA [2], computer vision-based (computer-based) [4], CAD [6], and Triple Negative Breast Cancer discrimination using MRI [7].

Training accuracy reveals the efficiency of an FPGA to accelerate the CNN model training computations, and its parallel processing ability improves training speed and accuracy compared to CPU/GPU training. Validation accuracy might lag behind training accuracy due to a complex model or insufficient regularization that overfits. FPGA-based systems speed up experimentation and hyperparameter tuning to discover the ideal validation accuracy configuration. Although GPUs allow parallel computation, they still struggle with huge models or datasets compared to FPGAs, which can be customized. Both CPUs and GPUs face memory bandwidth limitations for large-scale neural networks due to slow data transfers between the CPU/GPU and memory. Fig. (**13**) presents a graph drawn with the X-axis representing training accuracy and the Y-axis representing CNN systems, indicating that the proposed FPGA-based DnCNN evaluates accuracy more effectively in both training and validation than other non-FPGA-based CNNs.

This section examines the feasibility of a Support Vector Machine (SVM) for breast lesion classification. The SVM makes *a line* or *a hyperplane* that divides the data into classes. The process of making predictions based on massive datasets offers a high degree of accuracy by comparing the predicted class labels to the actual labels. For classification problems like distinguishing *benign* and *malignant* tumor types, the confusion matrix visualizes the success of ML models. The SVM classifier (classification report in Table **9**) for the proposed CNN model, *‘P-SVM’*, is trained on labeled data (*benign* and *malignant* tumor features) to find the best decision boundary (hyperplane) that separates the two classes. Once the model completes training, it can predict whether new data points are benign or malignant.

**Fig. (14):**
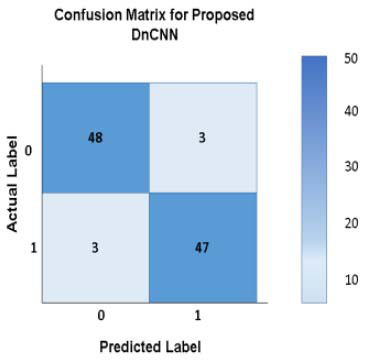
Confusion Matrix for DnCNN.

The confusion matrix aids in understanding the DnCNN’s fault behavior (whether it is confusing benign with malignant tumors or vice versa). For every prediction, the *P-SVM* produces a decision score [0 or 1], from which it can be interpreted into a class label (*malignant ‘1’ or benign ‘0’*). The confusion matrix contributes to improving the balance between false negatives and false positives, reducing the number of false negatives in identifying lesions. The matrix interpretation makes clear that only 3 misclassifications were found among 170 test samples. Thus, by analyzing the *‘P-SVM confusion matrix’* (Fig. **14**), the proposed DnCNN ultimately achieves better classification in distinguishing benign from malignant tumors.

The peak signal-to-noise ratio (PSNR) [29, 30] is a key factor in digital signal processing. A higher PSNR [31, 32] indicates less noise and distortion, making it a preferred metric. DSPs also use the Structural Similarity Index Metric (SSIM) [33, 34] as another qualitative metric. It measures the perceived change in structural information in a digital image, pixel-by-pixel when compared to the original image. Its values range from ‘0 to 1’, with ‘1’ being the most desired value. The proposed DnCNN PSNR and SSIM values against other state-of-the-art models from the literature, namely ADNet [29], BRDNet [30], complex-valued denoising [31] (CV-CNN), and BMCNN [32], are presented in Table **10**. From Table **10**, it is evident that the proposed DnCNN performs with better PSNR and SSIM than the state-of-the-art models.

## 5 FPGA IMPLEMENTATION

The proposed designs were developed using the ALTERA FPGA development kit, with the datasheet shown in Fig. (**15a**), and the breast cancer lesion segmentation along with its FPGA implementation are shown in Fig. (**15b**). As mentioned above in section 3.3.2, the P-SVM classification of decision score [0] as benign and [1] as malignant FPGA implementation is also shown in Fig. (**14**). The DE1 Altera development board comes with various peripherals that allow users to interact with the FPGA and test different types of circuits. A set of LEDs for output can be controlled for visual feedback, and multiple switches serve as input devices for the user to control the FPGA. The board features several push buttons for simple, user-driven inputs and often uses a 7-segment display to show numeric values or simple outputs, such as for debugging or showing the results of operations. For programming and communicating with the FPGA, a USB blaster is used to download the designs onto the board. To download the bitstream onto the board, there is no need to purchase an expensive programmer or special downloader.

## CONCLUSION AND FUTURE WORK

Image segmentation using a Convolutional Neural Network (CNN) with an optimized hybrid Vedic multiplier represents an advanced approach that aims to improve both the accuracy and efficiency of the model. The combination of CNNs for image segmentation and an optimized hybrid Vedic multiplier helps address the trade-off between accuracy and computational efficiency. This approach makes image segmentation more accessible for applications that require high performance and real-time processing, such as autonomous vehicles, medical imaging, and robotics. The incorporation of the hybrid Vedic multiplier optimizes the core operations of CNNs, ensuring the model remains both high-performing and resource-efficient, paving the way for more practical, scalable, and energy-efficient image segmentation solutions. The proposed DnCNN method outperforms conventional segmentation CNN models, improving the accuracy of segmentation by up to 96.3%. The proposed DnCNN approach improves precision while lowering computational complexity in terms of size and power consumption. It is very useful for early detection of female breast cancer. The proposed approach utilizes floating-point architecture based on Vedic hybrid multiplication and improves segmentation without increasing computing costs. Future segmentation architecture, design, and implementation can use Micro Service-Oriented Architecture. This will handle enormous quantities of numbers while using fewer resources and costs to ensure smooth health care, such as in dividing tasks or data. The limitation of the study is that larger image sizes increase processor size and gate count, affecting scalability.

## Figures and Tables

**Fig. (1):**
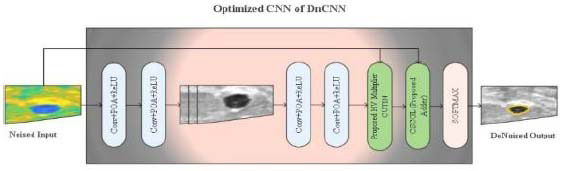
Functional flow of the proposed DnCNN (HV-FPGA) model.

**Fig. (2):**
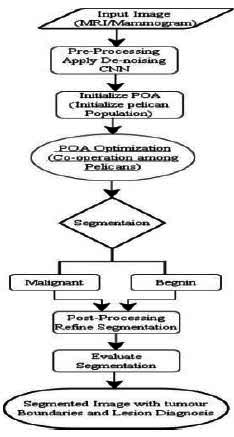
POA-DnCNN flow chart.

**Fig. (3):**
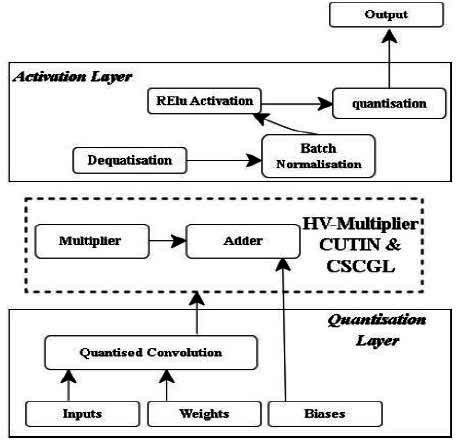
Process flow of a HV-multiplier.

**Fig. (5):**
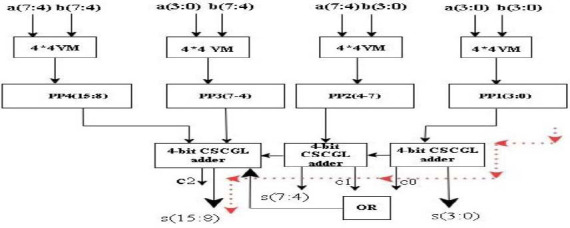
Execution of proposed HV-Multiplier.

**Fig. (6):**
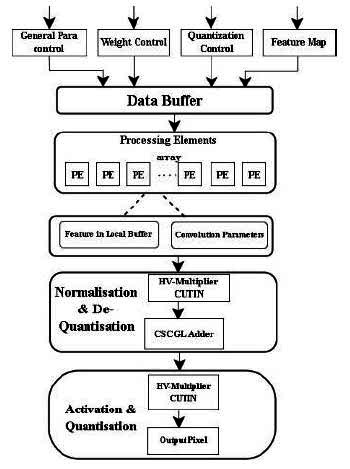
Components of quantized convolution.

**Fig. (8):**
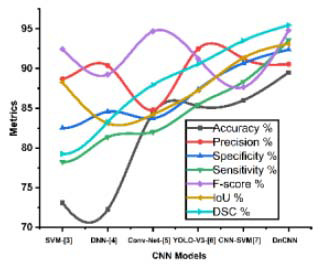
Analysis of segmentation metrics.

**Fig. (9):**
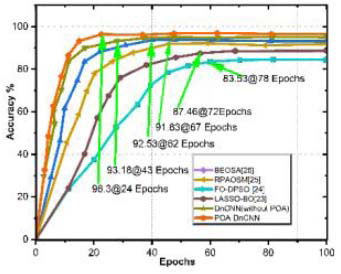
Optimization epochs.

**Fig. (10):**
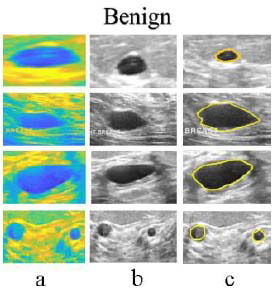
Benign: Segmented Results: (**a**) Image with Noise, (**b**) MATLAB Segmented Image, and (**c**) De-noised image for benign cancer lesions.

**Fig. (11):**
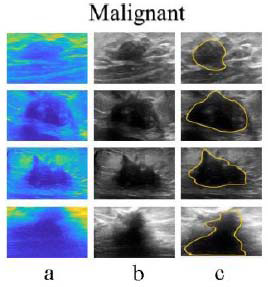
Malignant: Segmented results: (**a**) Image with noise, (**b**) MATLAB segmented image, and (**c**) De-noised image for malignant cancer lesions.

**Fig. (12):**
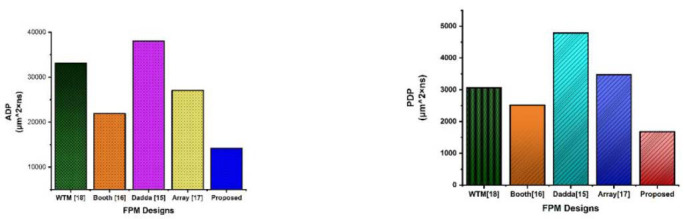
(**a**) ADP of Proposed HV-FPM against Existing FPM. (**b**) PDP of Proposed HV-FPM against Existing FPM.

**Fig. (13):**
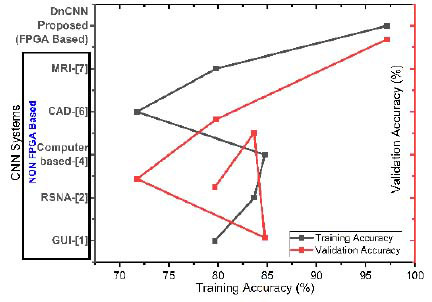
FPGA *vs*. non-FPGA-based CNN models.

**Fig. (15a):**
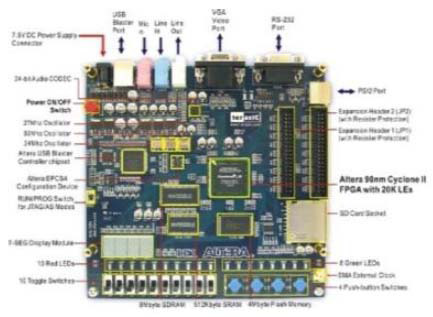
Altera data sheet.

**Fig. (15b):**
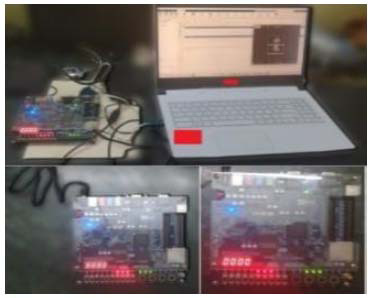
FPGA implementation.

**Table 1:** Comparison of existing multipliers.

** Reference **	** Multiplier Methods **	** Performance Evaluation **	** Remarks **
[15]	Dadda Multiplier	Area= 10678, power=1345, ADP= 38013 PDP= 2518	Irregular reduction stages result in high ADP.
[16]	Booth Multiplier	Area= 12672, power= 1456, ADP= 21922, PDP= 2518	High area leads to increased silicon cost.
[17]	Array Multipliers	Area= 11678, power= 1498, ADP= 27092, PDP= 3475	Higher power consumption increases energy costs, reducing overall system efficiency.
[18]	Wallace Tree (WT) Multiplier	Area= 13451, power= 1245, ADP= 33089, PDP= 3062	High ADP in WT leads to increased circuit complexity.
** Proposed HV-Multiplier **	Vedic Method	Area=**9872**, power=**1167**, ADP=**14215**, PDP=**1680**	Compared to existing multiplier designs, the HV-multiplier achieves lower critical values, making it ideal for breast lesion segmentation.

**Table 2:** Proposed model architecture for DnCNN.

Block	Layer	Filter Size and Mapping	Block	Layer	Filter Size and Mapping
Input	512×512×3	-	-	Conv_11	3×3,512
Block 1	Conv_1 Conv_2	3×3,64 3×3,64	Block 5	Conv_12 Conv_13	3×3,512 3×3,512
Block 2	Conv_3 Conv_4	3×3,128 3×3,128	-	Conv_14 Dropout-1	7×7,4096 d =0.5
Block 3	Conv_5 Conv_6 Conv_7	3×3,512 3×3,512 3×3,512	Block 6	Conv_15 Dropout-2	1×1,4096 d =0.5
Block 4	Conv_8 Conv_9 Conv_10	3×3,512 3×3,512 3×3,512	Final Map	Conv_16 SoftMax 256×256×3	1×1,2

**Table 3:** Segmentation performance of different DL models.

**State-of-art** **(from Literature)**	**Accuracy** **(%)**	**Precision** **(%)**	**Specificity** **(%)**	**Sensitivity** **(%)**	**F-score** **(%)**	**IoU** **(%)**	**DSC** **(%)**
SVM- [3]	73.1	88.67	82.52	78.23	92.46	88.25	79.26
DNN- [4]	72.25	90.35	84.56	81.35	89.24	83.1	83.25
Conv-Net- [5]	84.32	84.78	83.73	82.0	93.67	84.2	87.93
YOLO-V3- [6]	85.25	92.46	87.34	85.45	91.25	87.25	90.56
CNN-SVM [7]	86.0	91.26	90.67	88.34	87.65	91.32	93.50
**DnCNN** **(Proposed)**	**96.3**	**94.54**	**92.37**	**93.56**	**94.78**	**93.1**	**95.45**

**Table 4:** Device utilization summary of the proposed DnCNN for 8, 16, and 32 Bits.

**Device Utilisation**	**Utilised**			**Available**			**Utilisation**		
**8-Bits**	**16-Bits**	**32-Bits**	**8-Bits**	**16-Bits**	**32-Bits**	**8-Bits**	**16-Bits**	**32-Bits**
No. of Sliced LUTs	102	110	124	1,120,000	1,223,000	1,458,000	1%	1%	8%
No. of LUT Flip-Flops	103	119	135		_	_	_	_	_
No. of Occupied Cells	38	42	53	124,000	153,000	178,000	3%	3.4%	2.7%
No. of Bonded IOBs	28	36	78	500	800	1,400	5.6%	4.5%	5.5%

**Table 5:** Synthesis results comparison of conventional floating-point multiplier (FPM) architecture.

**FPM Architecture**	**TAD** **(ns)**	**Area** **(μ)**	**Power** **(μW)**	**PDP** **(μW×ns)**	**ADP** **(μm^2^×ns)**
Wallace Tree [18]	2.46	13,451	1,245	3,062	33,089
Booth [16]	1.73	12,672	1,456	2,518	21,922
Dadda [15]	3.56	10,678	1,345	4,788	38,013
Array-Based [15]	2.32	11,678	1,498	3,475	27,092
**Proposed (HV-Multiplier)**	**1.44**	**9,872**	**1,167**	**1,680**	**14,215**

**Abbreviations:** TAD - Time taken for the data to arrive, PDP = Power Delay Product, ADP = Area Delay Product.

**Table 6:** Comparison of device utilization of HV multiplier and existing multiplier design.

**Specifications**	**WT Multiplier** [18]	**Booth-Multiplier** [16]	**Dadda multiplier** [15]	**Array-Based** [17]	**HV-Multiplier** **(Proposed)**
No. of Occupied Cells	76	54	58	47	39
No. of IOBs	42	43	34	57	29
Propagation Delay (ns)	7.4	7.7	8.32	6.5	1.34
Time (ns)	8.3	6.7	7.8	5.75	2.34

**Table 7:** Comparison of hybrid vedic multiplier combined with different adders topology.

** Specifications **	** Hybrid Vedic Multipliers **			
	CPA [17]	CLA [15]	CSA [18]	** CSCGL ** ** Proposed **
No. of Sliced LUTs	125	162	125	**115**
No. of Occupied Cells	45	47	58	**41**
No. of Bonded IOBs	32	41	37	**32**
Propagation Delay (ns)	1.07	4.75	3.46	**0.378**
Power Consumption (Watts)	7.2	8.3	5.28	**1.07**

**Table 8:** Training and validation accuracy of dncnn against state-of-the-art.

CNN System	Training Accuracy (%)	Validation Accuracy (%)
FPGA Based	97.18	98.43
(1) DnCNN(Proposed)		
Non-FPGA Based	79.66	81.22
(1) GUI- [1]		
(2) RSNA- [2]	83.66	87.56
(3) Computer-based [4]	84.75	75.34
(4) CAD [6]	71.75	82.19
(5) MRI [7]	79.78	89.10

**Table 9:** Classification report.

** Classification **	** Predicted Benign **	** Predicted Malignant **
Actual Benign	True Negative (TN)	False Positive (FP)
Actual Malignant	False Negative (FN)	True Positive (TP)

**Table 10:** Image De-Noising Metrics.

**De-noising CNN models**	**PSNR (dB)**	**SSIM**	**SD (PSNR)**	**95% CI (PSNR)**	**SD (SSIM)**	**95% CI (SSIM)**	**Convergence Level**
ADNet [29]	32.41	0.76	0.45	[31.97 – 32.85]	0.02	[0.740 – 0.780]	Moderate
BRDNet [30]	34.56	0.872	0.5	[34.08 – 35.04]	0.015	[0.857 – 0.887]	Slow
CV-CNN [31]	33.14	0.651	0.4	[32.76 – 33.52]	0.025	[0.626 – 0.676]	Slow
BMCNN [32]	35.76	0.74	0.48	[35.29 – 36.23]	0.022	[0.718 – 0.762]	Moderate
DnCNN (Proposed)	39.25	0.954	0.35	[38.91 – 39.59]	0.01	[0.944 – 0.964]	fast

## Data Availability

The BUSI dataset is publicly available on Kaggle [28].

## LIST OF ABBREVIATIONS

DL: Deep learning
HV: Hybrid-Vedic
ML: Machine Learning
MRI: Magnetic Resonance Imaging
MACC: Multiply-accumulate
CAD: Computer Aided Diagnosis
DSC: Dice Similarity Coefficient
PE: Processing Element
